# Gluten‐free schooling: Navigating challenges and triumphs for children with celiac disease

**DOI:** 10.1002/jpr3.70013

**Published:** 2025-03-03

**Authors:** Vanessa Weisbrod, Nasim Khavari, Imad Absah, Dale Lee, Danny Mallon, Catherine Raber, Vahe Badalyan, Mary Shull, Ritu Verma, Ashley Dunn, Anava Wren, Farah Mardini, Lisa Fahey, Jocelyn Silvester, Tracy Ediger, Maureen Leonard, Javier A. Lopez‐Rivera, Hilary Jericho

**Affiliations:** ^1^ Celiac Disease Foundation Boston Massachusetts USA; ^2^ Stanford University Palo Alto California USA; ^3^ Mayo Clinic Rochester New York USA; ^4^ Seattle Children's Hospital Seattle Washington USA; ^5^ Cincinnati Children's Hospital Cincinnati Ohio USA; ^6^ Children's National Hospital Washington District of Columbia USA; ^7^ Children's Colorado Aurora Colorado USA; ^8^ University of Chicago Medicine Chicago Illinois USA; ^9^ Children's Hospital of Philadelphia Philadelphia Pennsylvania USA; ^10^ Boston Children's Hospital Boston Massachusetts USA; ^11^ Nationwide Children's Hospital Columbus Ohio USA; ^12^ Mass General Hospital for Children Boston Massachusetts USA

## Abstract

**Objectives:**

Celiac disease (CeD), an autoimmune disorder triggered by gluten ingestion, induces intestinal inflammation and varied symptoms. Treatment entails a strict gluten‐free diet (GFD), posing challenges for students, especially in schools with limited food choices. Nonadherence worsens symptoms, yet research on CeD's impact on students is scarce.

**Methods:**

The CeliacKIDS study, conducted across 11 United States academic medical centers, evaluated gluten exposure risk in pediatric CeD patients via a cross‐sectional survey from August 2020 to August 2021. Participants recruited from treating institutions were approved by respective Institutional Review Boards.

**Results:**

One hundred and sixty children aged 5–18 (65% female, 34% male, 1% other) participated. Only 12% had GF food options at school, 31% brought their own for celebrations, and 41% lacked gluten free (GF) snacks after school. Thirty‐six percent lacked a 504 plan, with 5% misinformed. Hand hygiene concerns included 24% using sanitizer and 10% rarely washing hands before eating. Sixty‐two percent disclosed CeD, 35% when prompted, and 3% refused, mainly 13‐year‐old males. Two percent hesitated to request GF options, and 2% consumed potentially gluten‐containing food from friends.

**Conclusion:**

Many US schools provide GF accommodations under the Americans with Disabilities Act (ADA) but lack national standards. Diverse GF options and education on GF‐safe practices are crucial for GFD adherence. Discrepancies in parent–child perceptions emphasize the need for better communication. Adolescents, particularly females aged 12–13 with 2+ years on a GF diet, face higher risks. Transparent family–school communication is vital for optimizing the school experience and ensuring GFD adherence. Comprehensive nationwide school training is essential for celiac patients' well‐being.

## Abstract (highlights)

**What is Known:**

- The only current treatment for patients diagnosed with celiac disease is strict adherence to a gluten‐free diet (GFD).
- Strict adherence to a GFD poses many social challenges at school.
- Variable availability of gluten‐free food options in schools and student mealtime behaviors increase their risks of gluten exposure, creating the potential for negative medical outcomes.

**What is New:**

- Continued adherence to the GFD is relatively high among elementary and secondary school students.
- There is substantial diversity and inconsistencies in what gluten free (GF) offerings, supports, and practices are present in public and private schools across the United States.
- Improved parent and child communication surrounding safe GF practices at school, greater availability and diversity of GF options at schools, as well as support resources for students are needed in public and private schools across the United States.

## 1 INTRODUCTION

Celiac disease (CeD) is a chronic autoimmune disease triggered by the ingestion of gluten (the major storage protein in wheat, barley, and rye) in genetically predisposed individuals, resulting in small intestine inflammation and a wide range of gastrointestinal and extra‐intestinal manifestations.^1^ Currently, a strict lifelong gluten‐free diet (GFD) is the only treatment for CeD. For students, maintaining this diet can be challenging, especially when school food options are limited, and deviations from the GFD can worsen symptoms and lower quality of life.

Under the Americans with Disabilities Act (ADA), CeD is recognized as a disability as it significantly impacts major life activities such as eating and digestion. Consequently, individuals with CeD are entitled to reasonable accommodations in schools, workplaces, and public spaces to ensure equal opportunities. A 504 Plan outlines necessary accommodations for students with disabilities, including those with CeD, addressing issues like the availability of gluten‐free (GF) options in cafeterias and at school events.^2^

There is limited research on the experiences of students with CeD and how these affect their adherence to the GFD. Our cross‐sectional survey study aims to assess the availability of GF foods, adherence to the GFD, and support resources for elementary and secondary students with CeD during the school day.

## 2 METHODS

This study utilized a cross‐sectional survey distributed across 11 US academic medical centers (Boston, MA; Washington, DC; Aurora, CO; Rochester, MN; Chicago, IL; Philadelphia, PA; Seattle, WA; Cincinnati, OH; Columbus, OH; and Palo Alto, CA) to families participating in the CeliacKIDS study, which evaluates the risk of gluten exposure in pediatric patients with CeD. Surveys were collected from August 2020 to August 2021. Participants were included if they had a confirmed diagnosis of CeD, with 88% diagnosed through biopsy, 5.7% meeting the European Society for Pediatric Gastroenterology Hepatology and Nutrition (ESPGHAN) criteria, and 6.3% diagnosed at the physician's discretion without biopsy or ESPGHAN criteria. Exclusion criteria included insufficient English proficiency, reliance on commercial GF formulas as the primary nutrition source, and any determination by the investigators that participation was inappropriate. The survey questions were crafted using simplified language for better readability (Supplemental Material 1). Children over 8 were instructed to complete the survey independently, while parents could assist children under 8. Participants were recruited via email or conventional mail from their treating institutions. Data analysis included descriptive statistics, qualitative feedback, and the calculation of discordance rates by comparing children's and parents' responses to each question, analyzed using a paired Wilcoxon signed‐rank test with R (version 4.3.3).

### 2.1 Ethics statement

This study received approvals from the Institutional Review Boards at Stanford University, Mayo Clinic, Seattle Children's Hospital, Cincinnati Children's Hospital, Children's National Hospital, Children's Colorado, University of Chicago Medicine, Children's Hospital of Philadelphia, Boston Children's Hospital, Nationwide Children's Hospital, and Mass General Hospital for Children. Since the participants in this study were minors, their guardians provided consent for their participation.

## 3 RESULTS

One hundred and sixty‐three elementary, middle, and high school aged children (18 years and younger) and their parents completed a survey developed by the study team. Mean age was 11.5 years (range 5–18), corresponding to 6th grade. One hundred and six children classified themselves as female (65%), 55 male (34%) and 2 (1%) other. Sixty‐seven (41%) children had been on a GFD for over 5 years, 86 (53%) for 1–4 years, and the remaining 10 (6%) for a period of less than 1 year. Although a free‐text question indicated that children attended a mix of private and public schools, the specific breakdown was not assessed, nor was specific grade level (Table 1).

**Table 1:** Patient demographics.

Demographics	*N* = 163 (%)
Age (years)	
0–5	1 (1)
6–10	69 (42)
11–15	74 (45)
16–20	19 (12)
Sex	
Female	106 (65)
Male	55 (34)
Other	2 (1)
Duration on the diet	
Not on the diet	0 (0)
Less than 1 month	0 (0)
1–3 months	1 (1)
4–6 months	0 (0)
7–12 months	8 (5)
1–4 years	86 (52)
5+ years	67 (41)
No response	1 (1)
Hospital location	
Boston, MA	38 (23)
Chicago, IL	14 (9)
Washington, DC	5 (3)
Philadelphia, PA	20 (12)
Cincinnati, OH	16 (10)
Aurora, CO	27 (17)
Rochester, MN	18 (11)
Columbus, OH	7 (4)
Seattle, WA	11 (7)
Palo Alto, CA	7 (4)

### 3.1 Availability of GF food in school (*N* = 160)

One hundred and twenty‐five (78%) students reported taking their own GF food to school, while only 12 (8%) ate GF food prepared in the school cafeteria. The remaining 14% ate a combination of GF food from home and school. Six percent of child and parental responses were discordant (*p* = 0.11).

### 3.2 School GF food offerings (*N* = 75)

Nineteen students (12%) reported that their school offered GF foods; 18 (11%) stated food was prepared in a shared kitchen with trained cooks to prevent cross‐contact; 16 (10%) mentioned prepackaged GF products; 12 (7%) indicated the school provided an advance menu with GF options for review; and 4 (2%) noted a buffet‐style meal with labeled GF items. Two children (1%) said their school had no GF offerings, and 1 (0.06%) reported not eating at school. Three students (2%) were homeschooled due to repeated gluten exposures. There was a 6% discordance in responses between children and parents (*p* = 0.74).

### 3.3 School celebrations (*N* = 160)

**Figure 1:**
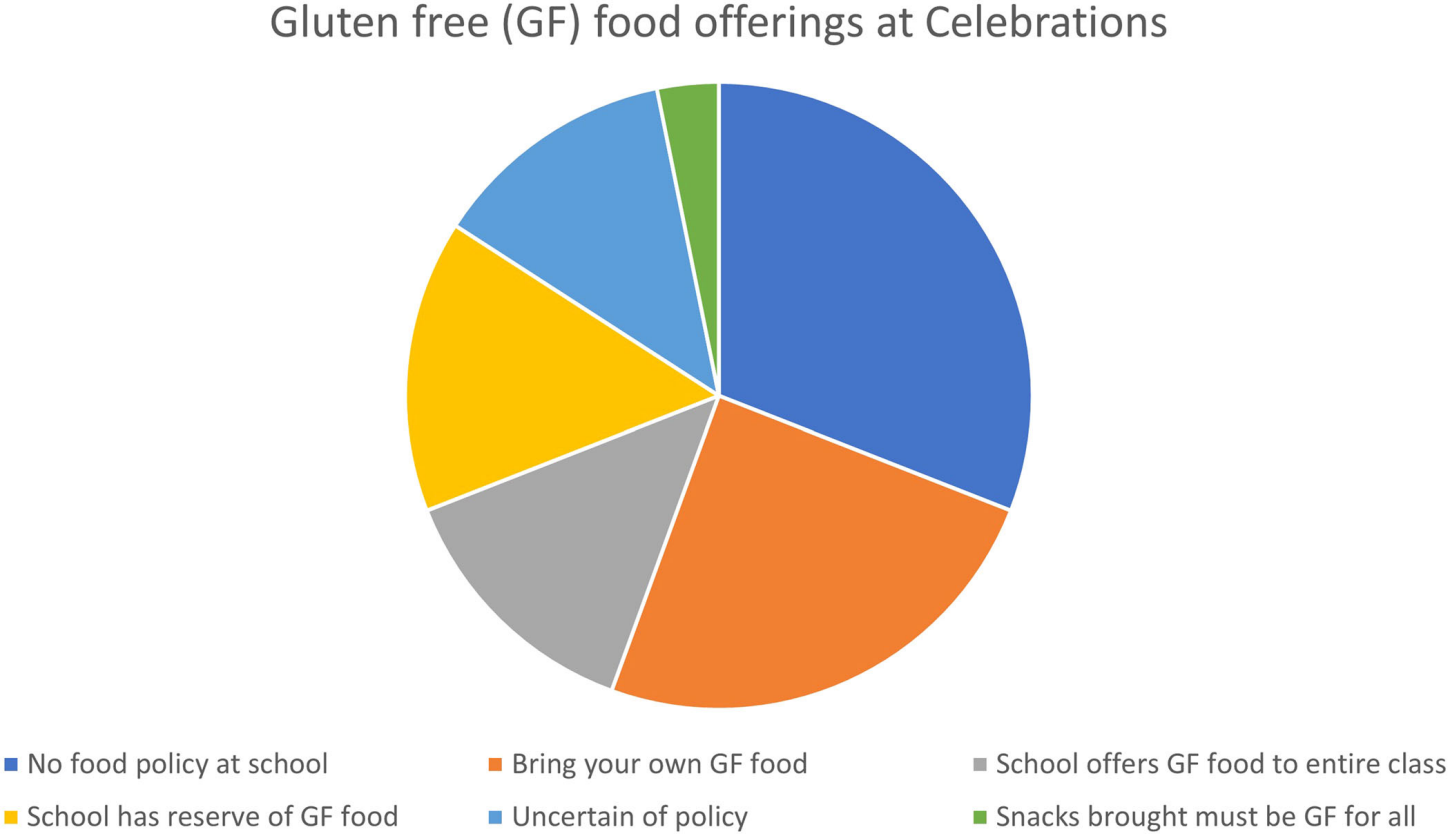
Gluten free food offerings at school celebrations.

Participants could select multiple responses. Sixty‐four students (39%) indicated a “no food” policy for celebrations; 50 (31%) were asked to bring their own GF items; 27 (17%) reported that the school provided a GF option for everyone; 31 (19%) noted a reserve of GF items for students; 6 (4%) stated that all snacks for class must be GF; and 26 (16%) were unsure of the policy. There was a 42% discordance between child and parental responses (*p* = 0.47) (Figure 1).

### 3.4 After school activities (*N* = 116)

Forty‐eight students (41%) reported no GF snacks available at after‐school activities, while 32 (28%) said GF snacks were available. Sixteen students (14%) indicated that GF snacks were supposed to be available but often were not, and 20 (17%) were unsure of the options. There was a 37% discordance between child and parental responses (*p* = 0.14).

### 3.5 504 plans (*N* = 152)

**Figure 2:**
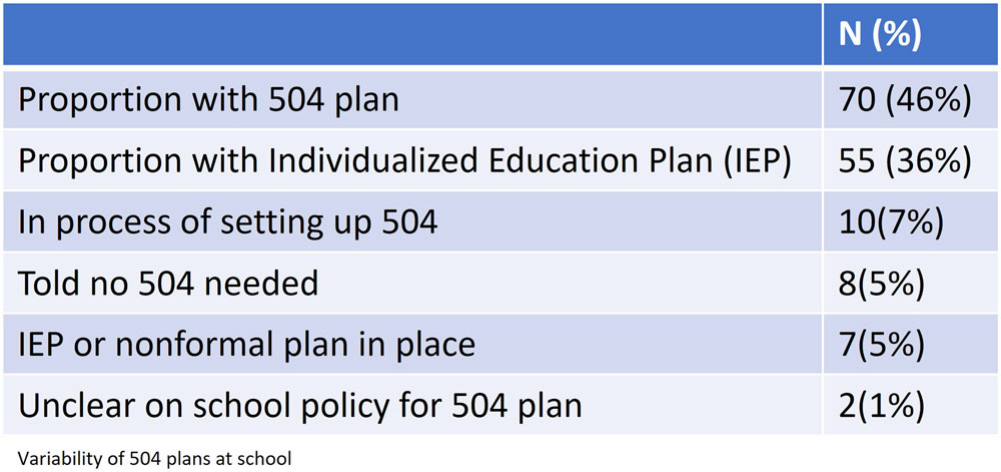
Variability of 504 plans at school.

Seventy students (46%) reported having a 504 plan for their CeD, while 55 (36%) did not. Ten students (7%) were in the process of establishing a 504 plan, 8 (5%) were informed that a plan was unnecessary, and 2 (1%) were unclear about the school's 504 policy. Additionally, five students (3%) mentioned having informal accommodations, and two (1%) had individualized education plans. There was a 22% discordance between child and parental responses (*p* = 0.34) (Figure 2).

### 3.6 Advocacy in the school environment (*N* = 162)

One hundred and one children (62%) indicated they will voluntarily announce their need for GF options at school, while 57 (35%) said they will do so only if asked. Four children (2%) responded that they would not announce their need for GF options, even when asked. There was an 11% discordance between child and parental responses (*p* = 0.38) (Figure S1).

### 3.7 Purchased food items at school (*N* = 161)

Sixty‐two children (39%) never purchased food at school. Among those who did, 78 (48%) exclusively bought GF items; six (4%) purchased GF items “much of the time,” six (4%) “some of the time,” five (3%) “a little of the time,” and four (2%) “none of the time.” There was an 18% discordance between child and parental responses (*p* = 0.19).

### 3.8 Sharing food (*N* = 162)

Eighty children (49%) stated they will never share a friend's food at school, while 78 (48%) will share only when they are certain it is GF. Four children (2%) said they would share even if unsure about GF status. This group consisted of 75% males, with a mean age of 13 years and an average of 4 years on the GFD. There was a 17% discordance between child and parental responses (*p* = 0.19).

### 3.9 Premeal hand hygiene (*N* = 160)

**Figure 3:**
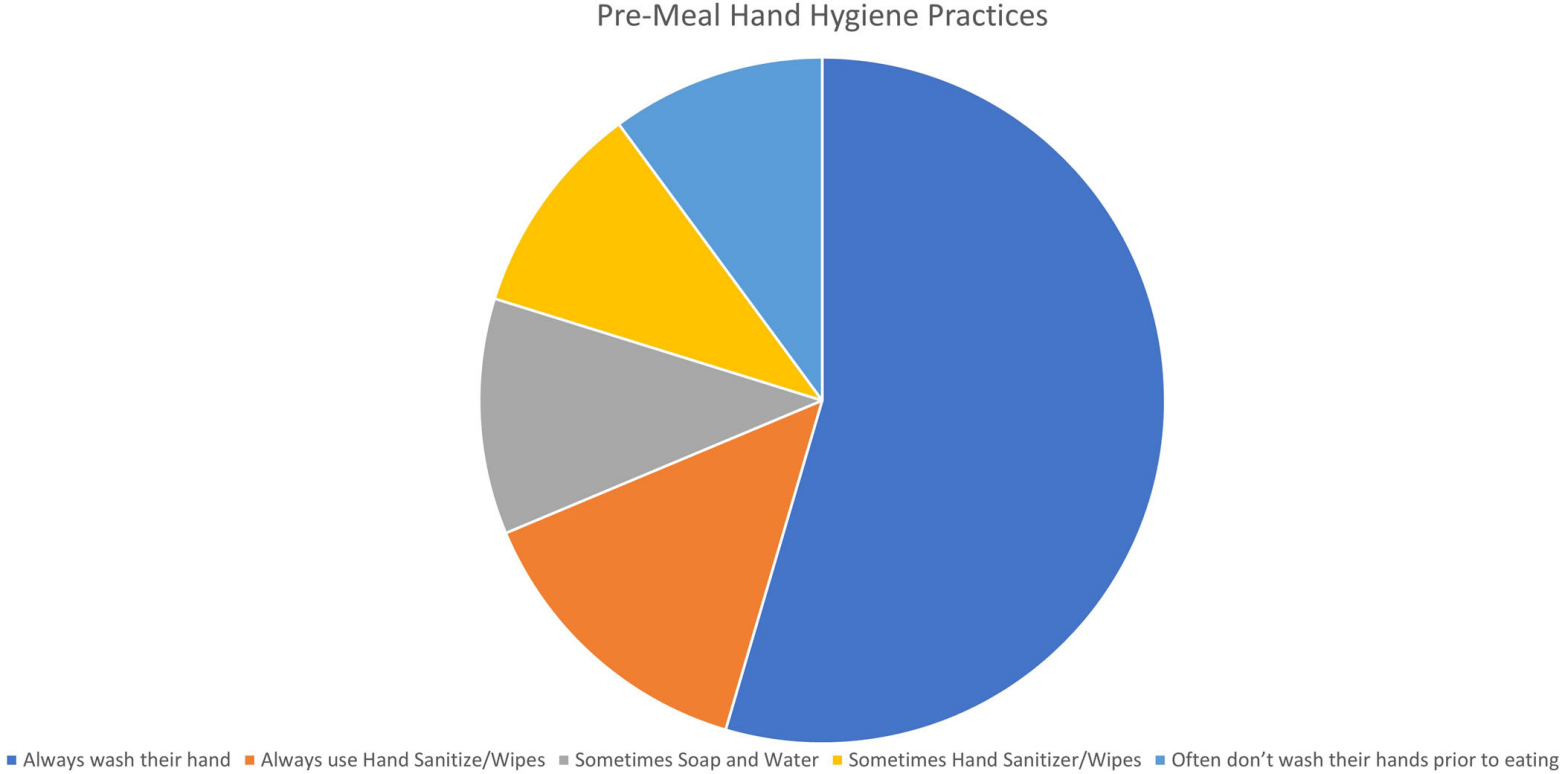
Premeal hand hygiene practices at school.

Eighty‐seven children (54%) always wash their hands with soap and water before eating at school, while 23 (14%) consistently use hand sanitizer or wet wipes. Eighteen children (11%) sometimes use soap and water, and 16 (10%) sometimes use hand sanitizer or wet wipes. Another 16 children (10%) often do not wash their hands before eating. This last group comprised 56% females, with a mean age of 13 years and an average of 3 years on the GFD. There was a 59% discordance between child and parental responses (*p* = 0.15) (Figure 3).

### 3.10 Hand hygiene following art supply use (*N* = 155)

Ninety‐one children (59%) always wash their hands with soap and water after using art supplies, while two (1%) use hand sanitizer or wet wipes. Twenty‐one children (14%) sometimes use soap and water, and five (3%) sometimes use hand sanitizer or wet wipes. Thirteen children (8%) often do not wash their hands. Additionally, 20 (13%) attend schools that only use GF art supplies, two (1%) bring their own GF supplies, and one (0.6%) leaves the classroom during art lessons involving gluten‐containing products. The group of 13 students who often do not wash their hands consisted of 62% females, with a mean age of 12 years and an average of 3 years on the GFD. There was a 48% discordance between child and parental responses (*p* = 0.14).

### 3.11 Gluten exposures at school: Intentional or accidental (*N* = 142)

Ninety‐two children (65%) reported never consuming gluten at school, while 29 (20%) consume gluten “a little of the time,” and 18 (13%) “some of the time.” Two children (1%) reported consuming gluten “much of the time,” and 1 (0.7%) “all of the time.” The last two categories consisted entirely of females, with a mean age of 12 years and an average of 2 years on the GFD. There was a 27% discordance between child and parental responses (*p* = 0.10).

## 4 DISCUSSION

Our descriptive study highlights the challenges faced by students with CeD in elementary and secondary education regarding the availability of GF foods, limited school support, high rates of risky behaviors, and intentional gluten consumption.

Findings indicate that only 12% of participants attended schools offering GF foods, 31% had to provide their own GF items for class celebrations, and 41% reported a lack of GF snacks at after‐school activities. Additionally, 36% did not have a 504 Plan, with 5% mistakenly believing it wasn't necessary. Concerning hand hygiene practices were observed, as 24% relied on hand sanitizer instead of handwashing, and 10% rarely washed their hands before eating, increasing their risk of gluten exposure. While 62% felt comfortable disclosing their CeD diagnosis at school, 35% would share it only when prompted, and 2% refused to disclose. Alarmingly, 2% hesitated to request GF options, and 2% admitted to consuming potentially gluten‐containing foods provided by friends, with intentional gluten consumption reported by 20% occasionally and 1% consistently.

Despite the GFD being the only current treatment for CeD, its adherence can disrupt social interactions and necessitate coping strategies as students learn to manage their condition. Numerous studies highlight the burden of a GFD on school‐age children, linking it to higher rates of depression, anxiety, social withdrawal, and feelings of isolation.^3^, ^4^, ^5^ Our findings are concerning, as existing laws meant to protect students with CeD and support their GFD adherence in schools are not being implemented in accordance with federal requirements.

CeD is considered a disability under the ADA and Section 504 of the Rehabilitation Act of 1973. These regulations state that individuals with physical or mental impairments substantially limiting major life activities are covered, including activities such as eating and digestion.^6^ The US Department of Justice has also confirmed that ADA compliance requires providing accessible food options for individuals with dietary restrictions.

In 2019, the Celiac Disease Foundation led a national initiative to develop the first recommendations for managing CeD in educational settings. These guidelines resulted from consensus meetings with representatives from 12 pediatric hospitals, national education organizations, school food service providers, public and private school administrators, and parents and children with CeD. Available at http://school.celiac.org, the recommendations offer crucial information for students, school administrators, nurses, and food service providers. They include the latest research to help schools implement safe practices, such as using gluten‐containing materials, substitution lists, and advice for home economics classes. The guidelines also address creating a 504 plan, providing CeD educational materials, increasing awareness of gluten exposure prevention, and enhancing communication between families and schools. Additional topics include safely using school supplies that may contain gluten and managing field trips, class celebrations, sports events, dances, catered activities, and other extracurriculars.^7^

Our study found that many public and private elementary and secondary schools in the United States provide some GF accommodations to comply with the ADA, but these accommodations are not standardized nationwide. There is a crucial need for increased availability and variety of GF options, as well as enhanced education on GF‐safe practices (e.g., preventing cross‐contact) to ensure consistent adherence to the GFD.

A notable finding was the discrepancy between children's and parents' perceptions of school GF practices. In several areas, parents reported more positive views than their children. For example, 5% more parents than children indicated that schools provided GF options for celebrations, 4% believed there was a plan for GF snacks at after‐school activities, and 6% thought their child freely shared their CeD diagnosis at school more than the children reported. However, these differences were not statistically significant.

Some surprising discrepancies arose regarding handwashing practices and gluten consumption. While only 3% of parents believed their child did not wash hands before eating at school, 10% of children admitted they did not. Again, these differences were not statistically significant. Additionally, nearly a quarter of children (24%) reported using hand sanitizer before eating at school, suggesting a lack of awareness about effective gluten prevention practices, as hand sanitizer does not remove gluten from hands.^8^

Encouragingly, most students (63%) reported washing their hands with soap and water before eating, surpassing parental estimates (52%). However, there were concerning discrepancies regarding gluten exposure: 4% more parents than children believed their child was never exposed to gluten. This gap may be due to children's reluctance to disclose gluten consumption for fear of negative consequences. Additionally, 2% of children admitted to frequent gluten consumption, a response not echoed by parents. This may underestimate the issue in the general population since our participants were recruited from large academic hospitals with specialized celiac centers and multidisciplinary teams. Despite these discrepancies, none were statistically significant.

Our study found that female students aged 12–13 who had been following a GF diet for over 2 years faced the highest risk of unsafe GF behaviors. This may be attributed to developmental factors, as adolescents in this age group typically seek more independence, making it harder to consistently adhere to safe GF practices while navigating social pressures to fit in. Additionally, transitions to middle school or other educational changes could create new challenges in accessing GF options and managing dietary needs in unfamiliar settings. Further research on the specific obstacles faced by this demographic can help develop targeted interventions and support strategies.

Our study reveals that while elementary and secondary schools may offer GF options for students with CeD, there often exists a lack of experience and understanding regarding their specific needs. Both the quantitative and qualitative data—gathered from participants' thoughtful responses to open‐ended questions (Supplemental Material 2)—offer valuable insights for families and schools on how to enhance the educational experience for these students. Furthermore, the findings underscore existing gaps and suggest next steps for improving the dissemination of national recommendations that may not yet be fully utilized.

Our study shows that although elementary and secondary schools may provide GF options for students with CeD, there is often a lack of understanding of their specific needs. The quantitative and qualitative data, including participants' responses to open‐ended questions, provide valuable insights for families and schools on how to improve the educational experience for these students. Additionally, the findings highlight existing gaps and recommend steps to better disseminate national guidelines that may not be fully utilized.

While this study provides valuable insights into the challenges faced by students with CeD in schools, several limitations must be acknowledged. First, the small sample size and absence of a control group limit the generalizability of our findings, despite data being collected from various US school settings. Future research should include larger, more diverse participant pools to better understand GFD adherence in elementary and secondary schools. Second, the use of a nonstandardized survey restricts the conclusions that can be drawn. Future studies should employ validated survey instruments and qualitative methods to improve data collection reliability. Third, the lack of information on whether children attended public or private schools limits our understanding of safe GF practices, hindering targeted interventions in specific educational contexts. Lastly, self‐selection bias may affect our study design, as responders and nonresponders may have significantly different experiences.

Ensuring adherence to a GFD and cultivating safe habits will require significant collaboration among various stakeholders. Future research should focus on mixed‐method approaches, including validated surveys and qualitative interviews or focus groups, to provide data that informs public and private elementary and secondary schools on effectively supporting students with CeD and capturing the diverse experiences of students, parents, and educators. Additionally, there is a pressing need for ongoing community‐based participatory research that involves stakeholders such as school superintendents, parents, children, teachers, and healthcare professionals. Collaborating with these groups can facilitate the codesign and implementation of interventions that promote GFD adherence and support students with CeD, ultimately enhancing their academic success and quality of life.

## 5 CONCLUSION

This cross‐sectional survey study identified factors influencing the experiences of elementary and secondary school students with CeD, along with the challenges and strategies they employ to maintain adherence to a GFD. Our findings provide valuable insights to enhance the success, quality of care, and GFD adherence among these students. Future research should include a larger sample of CeD patients to improve statistical power and allow for causal inferences on factors affecting GFD adherence. Including comparison groups of similarly aged students without CeD will also strengthen the validity of these findings and help identify the unique challenges faced by students with CeD. Moreover, adopting mixed‐method approaches and community‐based participatory strategies will highlight the perspectives of those directly impacted by CeD. Collaborating with stakeholders will allow researchers to create evidence‐based interventions tailored to the specific needs of students with CeD, ultimately supporting their academic success and quality of life.

## CONFLICT OF INTEREST STATEMENT

The authors declare no conflict of interest.

## Supporting information

Supplemental Material 1 – Child School Survey.

Supplemental Material 2 – Challenges Faced by Students at School.

Supplemental Figure 1 – Advocacy in School Environment.
