# Supplementary material for: Gluten‐free schooling: Navigating challenges and triumphs for children with celiac disease
Source: JPGN Rep. 2025 Mar 3;6(2):99–106. doi: 10.1002/jpr3.70013 (PMC12078044; doi:10.1002/jpr3.70013)
Supplement: Supplementary file 1 — Supplemental Material 1 – Child School Survey. [file JPR3-6-99-s001.docx]

Child Survey

How old are you? [free text]

Please specify your sex.

- Male
- Female
- Other

How long have you been trying to follow a gluten-free diet?

- I am not on a gluten-free diet
- Less than 1 month
- 1-3 months
- 4-6 months
- 7-12 months
- 1-4 years
- 5+ years

How do you handle food for school?

- I take my own food
- The school provides food
- Combination: some food from home and some food from school
- Other [please explain]

If your SCHOOL PROVIDES FOOD, how is gluten-free food served? Select all that apply.

- Not applicable – school does not serve gluten-free food
- Serves pre-packaged gluten-free labeled products
- Oﬀers naturally gluten-free foods
- Cooks gluten-free food items in a shared kitchen that has been trained on preventing cross- contact
- Gluten-free packaged items kept separate and prepared in a dedicated gluten-free space
- Provides gluten-free menu options in advance for parent/student review
- Buﬀet-style food service with labeled gluten-free options
- Other [please explain]

What does your school do for special celebrations like birthdays and holidays? Select all that apply.

- No food celebration policy
- Provide a gluten-free option
- I am asked to provide a gluten-free option
- If a student brings food to share with classmates, then it must be gluten-free as well as free of

other allergens in the class/school

- School stores gluten-free items for me to have during special events
- I don’t know
- Other [please explain]

Is there a plan in place to provide gluten-free food and/or snacks for after-school activities?

- No
- Yes, there is a plan, but often gluten-free food is not available
- Yes
- Not applicable – I do not participate in after-school activities
- I don’t know

Do you have a formal accommodation plan for celiac disease and the gluten-free diet at school? (e.g., 504 plan or Individual Health Plan)

- Yes
- Not yet, but I would like to set one up
- We discussed with the school, but they said that I don’t need one
- No
- I don’t know
- Other [please explain]

Do you make your gluten-free needs known to adults/school staﬀ?

- Yes, I tell them that I need gluten-free food without being asked
- Yes, I will tell them that I need gluten-free food if I am asked
- No, I do not tell others I need gluten-free food, even if asked
- No, I can not tell people that I need gluten-free food

How often do you make gluten-free choices if you buy food on your own outside of the school cafeteria (e.g., the corner store, vending machine, restaurant with friends)?

- Not applicable – I don’t buy food on my own
- Not applicable – I am not on a gluten-free diet
- All of the time
- Much of the time
- Some of the time
- A little of the time
- None of the time
- I don’t know

Do you share food with friends?

- Yes, even if I am not sure that the food is gluten-free
- Yes, but only if I am sure that the food is gluten-free
- No
- I don’t know
- Other [please explain]

Do you wash your hands BEFORE eating at school?

- Yes, always with soap and/or water
- Yes, always with hand sanitizer or wet wipes
- Sometimes with soap and/or water
- Sometimes with hand sanitizer or wet wipes
- No, I don’t usually wash my hands
- I don’t know
- Other [please explain]

Do you wash your hands AFTER using art materials at school (e.g., papier-mâché, ingredients for cooking class, modeling dough)?

- Not applicable – My school does not use gluten-containing materials
- Yes, always with soap and/or water
- Yes, always with hand sanitizer or wet wipes
- Sometimes with soap and/or water
- Sometimes with hand sanitizer or wet wipes
- No, I don’t usually wash my hands
- I don’t know
- Other [please explain]

How often do you think you are exposed to gluten during extracurricular activities, sports or after-school programs either accidentally or by choosing to eat foods with gluten?

- All of the time
- Much of the time
- Some of the time
- A little of the time
- None of the time
- I don’t know
