# Supplementary material for: Gluten‐free schooling: Navigating challenges and triumphs for children with celiac disease
Source: JPGN Rep. 2025 Mar 3;6(2):99–106. doi: 10.1002/jpr3.70013 (PMC12078044; doi:10.1002/jpr3.70013)
Supplement: Supplementary file 2 — Supplemental Material 2 – Challenges Faced by Students at School. [file JPR3-6-99-s003.docx]

**Supplemental Material:**

**Challenges Faced by Students at School:**

During the survey, both children and parents were given the opportunity to offer free-text responses to the questions. Presented below are selected responses from participants regarding challenges encountered at school.

*“It just sometimes is hard as a teen in social situations to always have to have my own food, like when going out to eat, staying at friends' houses, school events etc.”*

*“It is difficult to explain Celiac to my teachers and sometimes* people *think that I am just doing it for attention.”*

*“Feeling left out from peers”*

*“Not having food that other people can--no school lunches, nothing at friends' birthdays, parties at church and after school events.”*

*“I wish I could eat foods that other kids could eat. I wish I could eat school food like the other. I wish I could eat out easier while traveling or with friends. I wish the prices were the same.”*

*“Can be hard to participate in social events around eating with strict diet restrictions and the possibility of cross contamination is stressful.”*
