# Supplementary figures and images for: Gluten‐free schooling: Navigating challenges and triumphs for children with celiac disease
Source: JPGN Rep. 2025 Mar 3;6(2):99–106. doi: 10.1002/jpr3.70013 (PMC12078044; doi:10.1002/jpr3.70013)

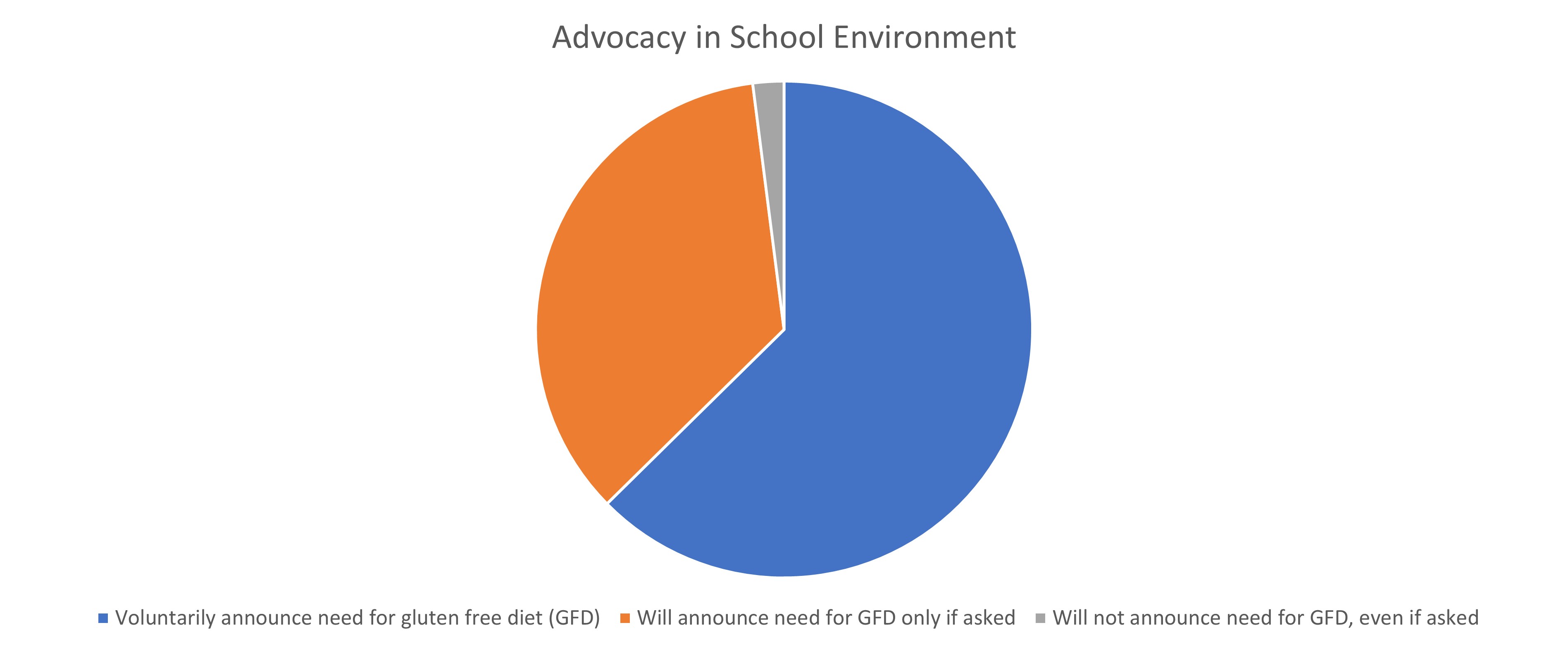

Supplement: Supplementary file 3 — Supplemental Figure 1 – Advocacy in School Environment. [file JPR3-6-99-s002.jpg]
